# The Potential Role of Gut Mycobiome in Irritable Bowel Syndrome

**DOI:** 10.3389/fmicb.2019.01894

**Published:** 2019-08-21

**Authors:** Yu Gu, Guoqiong Zhou, Xiali Qin, Shumin Huang, Bangmao Wang, Hailong Cao

**Affiliations:** Department of Gastroenterology and Hepatology, Tianjin Medical University General Hospital, Tianjin, China

**Keywords:** gut mycobiome, irritable bowel syndrome, inflammation, visceral hypersensitivity, gut-brain axis, fungal-bacterial interactions

## Abstract

The human gut is inhabited by diverse microorganisms that play crucial roles in health and disease. Gut microbiota dysbiosis is increasingly considered as a vital factor in the etiopathogenesis of irritable bowel syndrome (IBS), which is a common functional gastrointestinal disorder with a high incidence all over the world. However, investigations to date are primarily directed to the bacterial community, and the gut mycobiome, another fundamental part of gut ecosystem, has been underestimated. Intestinal fungi have important effects on maintaining gut homeostasis just as bacterial species. In the present article, we reviewed the potential roles of gut mycobiome in the pathogenesis of IBS and the connections between the fungi and existing mechanisms such as chronic low-grade inflammation, visceral hypersensitivity, and brain-gut interactions. Moreover, possible strategies targeted at the gut mycobiome for managing IBS were also described. This review provides a basis for considering the role of the mycobiome in IBS and offers novel treatment strategies for IBS patients; moreover, it adds new dimensions to researches on microorganism.

## Introduction

Fungi are ubiquitous microbes existing in diverse environments and are indispensable members of human intestinal ecosystem. Commensal fungi are much less studied than bacteria, as fungi constitute a tiny fraction of the symbiotic microbes in humans and most of them are unculturable. Despite this, steady work has been carried out to explore this mysterious organism in recent years. The composition of fungi on the skin, oral, airway, genitourinary tract, and gastrointestinal tract has been sequenced through culture-dependent or -independent methods (Huffnagle and Noverr, 2013). Some possible factors influencing the colonization of fungal microbiota such as genetic factors, diet, and immune response, are reported (Cui et al., 2013). Gut mycobiome is receiving increasing research interests due to its potential associations with various digestive diseases, including inflammatory bowel disease (IBD), IBS, colorectal cancer (CRC), and so on (Bożena et al., 2016). Among the studies of fungi- and gut-associated diseases, most frequently explored is IBD (Richard et al., 2015; Sartor and Wu, 2017; Sokol et al., 2017). Instead, only a few articles reported that this eukaryotic microbe or its metabolites have associated with irritable bowel syndrome (IBS) and how they interact with one another is still largely unknown.

IBS is one of the most common functional gastrointestinal diseases with high prevalence worldwide. According to Rome IV, IBS is defined as a symptom-based disorder with the presence of recurrent abdominal pain associated with defecation or changes in bowel habits. (Ford et al., 2017) In recent decades, IBS has gained extensive attention owing to its increasing incidence and various detrimental effects on human health. This chronic functional disease was reported to influence 7–21% of the global population (Lovell and Ford, 2012), and the prevalence in Asia is approximately 10% (Sperber et al., 2017). IBS is classified into four subtypes, named as IBS with constipation (IBS-C), IBS with diarrhea (IBS-D), mixed IBS (IBS-M), and unsubtyped IBS (IBS-U; Longstreth et al., 2006; Mearin et al., 2016). The subtyping of IBS provides guidance for therapeutic strategies. However, the clinical classification is based on the various symptoms self-reported by patients, which may result in the discrepant or weak therapeutic efficacy. Emerging evidence proposes that managements targeted at pathophysiologic mechanisms could be more effective and economical (Chang and Talley, 2010; Mayer et al., 2015; Holtmann et al., 2017).

**Figure 1:**
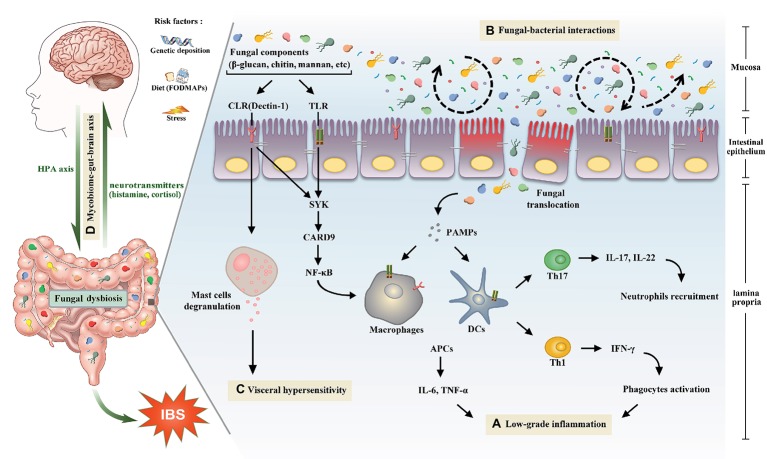
The potential role of gut mycobiome in the pathogenesis of irritable bowel syndrome. Gut mycobiome is involved in the existing mechanisms of irritable bowel syndrome including low-grade inflammation, intestinal dysbiosis, visceral hypersensitivity, and brain-gut interactions. **(A)** Pattern recognition receptors of human immune system, including CLRs and TLRs, can recognize fungal components such as β-glucan, chitin, and mannan, which will drive a series of downstream signaling pathways, then cause proinflammatory reactions and lead to low-grade inflammation in the intestine. **(B)** Tight interactions between fungi and bacteria are observed in the intestinal microecosystem. Fluctuations of either of the two species will lead to the intestinal dysbiosis. **(C)** Recognition of β-glucan by CLRs can trigger mucosal mast cells degranulation, which is a crucial mechanism of visceral hypersensitivity. **(D)** Intestinal mycobiome is another essential member of brain-gut axis aside from bacteria. Central nervous system can influence the composition of gut mycobiome through the HPA axis, which is closely associated with cognitive functions and gastrointestinal dynamics and colonic hypersensitivity. In turn, the mycobiome can release neuromediators such as cortisol and histamine to react on the central nervous system. Abbreviations: CLRs, C-type lectin receptors; TLRs, Toll-like receptors; SYK, spleen tyrosine kinase; CARD9, caspase recruitment domain family, member 9; NF-κB, nuclear factor-kappa B; HPA, hypothalamic-pituitary-adrenal; PAMPs, pathogen-associated molecular patterns; DCs, dendritic cells; APCs, antigen presenting cells; IL, interleukin; TNF, tumor necrosis factor; Th, T helper cell; IFN-γ, interferon gamma.

As a multifactorial disease with no single-disease model could entirely explain it, the precise mechanisms of IBS are still far from being completely understood (Oświęcimska et al., 2017). Several possible pathways referring to the initiation and progression of IBS symptoms have been identified, including psychological stress (Chang, 2011; Qin et al., 2014), infection/inflammation, antibiotics exposure (Ianiro et al., 2016; Klem et al., 2017), immune dysfunctions (Spiller and Garsed, 2009), abnormal brain-gut axis (Fichna and Storr, 2012; Jin et al., 2016; Moloney et al., 2016), and altered gut microbiota (Ringel-Kulka et al., 2015; Raskov et al., 2016). Genetic predisposition (Gazouli et al., 2016) and some environmental factors (e.g., diet and pollution; Lackner et al., 2014; Gibson et al., 2015; Marynowski et al., 2015) have also been revealed to be associated with IBS onset. One of the important causes for IBS is intestinal dysbiosis, which has drawn extensive attention and become a focal realm in the researches of gastroenterology. The composition of gut microbiota is proved to be evidently different in IBS patients relative to healthy individuals. Although a great number of studies on the contribution of intestinal microbiota to IBS emerge rapidly, nearly exclusive focus of intestinal microbiota is given on the bacterial species. Conversely, the role of gut mycobiome is underestimated. In this article, possible roles of mycobiome acting in the pathogenesis of IBS were provided (Figure 1), and potential mycobiome-directed therapeutic strategies for IBS were also described.

## Gut Mycobiome

Gut mycobiome is a general designation of intestinal fungi and their collective genome. Actually, the mycobiome inhabits not only gastrointestinal tract but also skin, respiratory tract, genitourinary tract, and other mucosal surfaces in the human host. The microorganisms in human body contain bacteria, archaea, fungi, and virus, and the number of microbial cells is estimated up to 10^14^, which is 10 times over the number of human cells (Cani, 2018). The gastrointestinal tract is the most heavily colonized organ; moreover, the colon alone covers more than 70% of the whole microorganisms in humans (Ley et al., 2006; Sekirov et al., 2010). Compared to the great number of bacterial microbes, fungi were reported to make up fewer than 0.1% of microorganisms in gastrointestinal tracts (Qin et al., 2010). With the development of microbial detection methods, our understanding of fungal microbes is progressing forward step by step. Initially, fungi were detected based on the traditional culture-dependent methods such as growth on media, microscopic observation, and biochemical analysis. Whereas, only a few of fungal species can be detected due to the fact that most of them are non-culturable. Applications of molecular techniques detecting microbes without the need of cultivation immensely boost the exploration of novel species in fungal communities. In recent years, next generation sequencing, which is also called high-throughput sequence, has been widely employed in microbiome detection. Similar to 16 s rRNA sequencing for bacteria, fungal spectrum as well can be reestablished through RNA sequencing. The most applied techniques for detecting fungi are 18S rRNA and internal transcribed spacer (ITS) sequencing. 18S rRNA sequencing can identify fungi at species level, and the highly conserved regions reflect phylogenetic relationships among species. ITS regions include ITS1 located between 18S and 5.8S genes and ITS2 between 5.8S and 28S rRNA. As ITS regions are not part of highly conserved areas of the ribosomal DNA, the variability of ITS is greater than 18S rRNA. Therefore, ITS regions are divergent enough among fungi to characterize fungal strains at species and even subspecies level. Actually, of the two methods mentioned above, which one is more suitable for detecting gut mycobiome still remains controversial.

The ITS region has been considered as a universal marker for fungal community identification. It has been utilized in a number of researches for decades, partly because the public databases for this target are available (Tang et al., 2015). However, a recent retrospective study compared 18S rRNA screening to ITS amplicon sequencing and culture and proposed an opposite point. Result of this study showed that 18S rRNA sequencing detected fungal pathogens in 10 ITS negative samples and declared that 18S rRNA RT-PCR combined with SANGER sequencing has a higher sensitivity and a shorter processing time than ITS sequencing (Wagner et al., 2018). To some extent, combination of 18S rRNA and ITS may be a better choice for fungal detection and classification. Results of the existing methods vary from one another, and no consensus on the best methodology for identifying gut mycobiome has yet been reached.

According to the present databases, the gut mycobiome can be detected in around 70% of the population, and the intestines harbor at least 267 fungal species (Schulze and Sonnenborn, 2009; Suhr and Hallen-Adams, 2015). Plenty of evidence proposed that the fungi in human gut mainly annotated to three phyla, including *Ascomycota*, *Basidiomycota,* and *Zygomycota* (Hoffmann et al., 2013; Gouba and Drancourt, 2015; Mar et al., 2015; Suhr and Hallen-Adams, 2015). Hoffmann et al. reported 66 fungal genera in fecal samples from 96 healthy individuals. *Saccharomyces* was found to be the most prevalent genus (present in 89% of tested samples), followed by *Candida* and *Cladosporium*, present in 57 and 42% of the specimens, respectively (Hoffmann et al., 2013). Another study using ITS-based sequencing reported *Penicillium* to be the most prevalent genus, followed by *Candida* and *Saccharomyces* (Mar et al., 2015). Whereas, the recent analytic data of stool samples from the Human Microbiome Project (HMP) cohort revealed the dominated genera in healthy human feces were *Saccharomyces*, *Malassezia*, and *Candida* (Nash et al., 2017). As seen above, copious fungi are existent in the human digestive tract, whereas no distinct and valid consensus on fungal repertoire has been reached. What is more, it is hard to say that the existing data for intestinal mycobiome are rigorously reliable. As some consistently detected fungi are presumed to be environmental origin, it is difficult to distinguish these fungal species are truly colonizing in the intestine or just transient members originating from environment and diet. A study in 2018 compared the ITS2 sequencing results of HMP stool samples with healthy controls. Results showed that *Saccharomyces cerevisiae* turned undetectable in fecal samples when a *Saccharomyces cerevisiae*-free diet was adopted. In addition, *Candida albicans* was greatly reduced to a lower level after a higher frequency of teeth cleaning. In this study, investigators claimed that the fungal microbes found in stools from healthy adults can be explained as dietary or oral derivation, and no fungi are routine colonizers in the healthy intestine (Auchtung et al., 2018). It follows that given the limited detection methods and extremely low abundance, the composition of gut mycobiome still requires further verification.

Aside from the species of fungi harbored in human gastrointestinal tract, factors influencing the constitution of intestinal fungal have also been explored. The influencing factors include host genotype, physiology (e.g., sex and age), life style, and environmental factors like geographical location and so on (Angebault et al., 2013; Cui et al., 2013). Dietary mode is a crucial factor affecting the intestinal fungal composition, due to that food ingestion is the main approach for microorganisms entering the digestive tract. It was previously reported by Hoffmann and colleagues that abundance of *Candida* was positively related to recent carbohydrate intake and negatively associated with saturated fatty acid (Hoffmann et al., 2013). What is more, fungal composition in human gut was found to be considerably different between individuals with diverse dietary patterns. For instance, the levels of *Fusarium*, *Malassezia*, *Penicillium,* and *Aspergillus* in vegetarians were reported to be much higher than people with Western diet (Hallen-Adams and Suhr, 2017).

Anyway, the same purpose of various studies on gut mycobiome is to inquire the implications that fungal microbes exert on human health. Evidence has revealed that disturbed intestinal fungi community occurred in diverse digestive diseases, including IBD, CRC, IBS, and so on. Take CRC as an example, a multicenter clinical practice based on 783 individuals revealed fecal fungal dysbiosis in CRC patients. An increased Basidiomycota/Ascomycota ratio and enriched class Malasseziomycetes with decreased Saccharomycetes and Pneumocystidomycetes were identified in CRC fecal samples (Coker et al., 2019). A few clues proving the associations between fungi and IBS were also reported and listed as follows. Decreased mycobiome diversity was found in IBS patients, while in maternally separated rats, increased α-diversity of mycobiome and fungal dysbiosis were observed (Botschuijver et al., 2017, 2018). Besides, autism patients with constipation as well took on aberrant composition of gut mycobiome (Strati et al., 2017). Furthermore, a commonly used yeast probiotics, *Saccharomyces boulardii*, was found able to reverse gastrointestinal dysfunction in IBS mice model and improve the quality of life in IBS patients (Abbas et al., 2014; Brun et al., 2017). Another instance is that foods that contain more yeast are more likely to induce IBS-specific symptoms (Costabile et al., 2014). The symptoms of IBS were considered possibly generated from *Candida* overgrowth (Nahas, 2011). Moreover, there were also a handful of studies showing that fungal components or products could bring about IBS-related manifestations in sensitive individuals (Santelmann and Howard, 2005). In spite of these evidence listed, the specific mechanisms of the mycobiome affecting IBS yet remain to be illustrated. This is a subject need to be explored in detail, which will supplement evidence to the interactions of intestinal microecology and human health.

## Irritable Bowel Syndrome

As a prevalent functional bowel disorder, IBS may not be lethal to most patients, whereas it significantly increases the socioeconomic burden and reduces the quality of life of sufferers (Canavan et al., 2014). Thus, accurate diagnosis and efficacious interventions are of great significance. Due to be traditionally regarded as a functional disease with no structural or biochemical lesions, IBS is diagnosed based on its chronic and recurrent symptoms, and the aims of treatments are mainly to relieve the symptoms. However, symptoms of IBS are frequently atypical and may transform over the course of disease. Studies have justified that nearly half of IBS patients had changed symptoms over time (El-Serag et al., 2004), and patients may transfer between subtypes (Engsbro et al., 2012). Hence, diagnosis and therapies of IBS relying on symptoms are obviously deficient. Along with increasing importance attached into the pathogenesis of IBS, the theory that IBS is a somatosensory disorder without structural abnormalities has been challenged. In addition, the interventions based on pathophysiological mechanisms are supposed to have considerable benefits.

Among the various causal factors for IBS, the role of intestinal micro dysbiosis is most frequently described. In more than 70% of IBS patients, a reduced diversity of microbiota is demonstrated, and the composition of gut microbiota shows significant difference from healthy controls (De Palma et al., 2014; Raskov et al., 2016). The common findings of microbiota alternation in IBS are an increase in facultative anaerobic bacteria like Streptococcus spp. and a reduced abundance of *Lactobacilli* and *Bifidobacteria* (Lee and Lee, 2014). An elevated ratio of fecal *Firmicutes*/*Bacteroidetes* in IBS patients is as well widely accepted in plentiful studies (Ohman and Simrén, 2013). Nevertheless, the results of studies on the microbiota in IBS are inconsistent. Based on the data to date, only a subgroup of IBS patients demonstrated compositional alternations. Frost et al. reported that fecal microbiota of healthy controls and individuals with functional abdominal pain did not show any significant differences (Frost et al., 2019). Another study analyzing the fecal and mucosal microbiota also reached the analogous conclusion that the similar composition of microbiota in IBS-D and controls could not explain the pathophysiological states and manifestations of IBS-D (Maharshak et al., 2018). Phenotypic variations, small sample sizes, and different designs might all lead to the fact that compositional changes of microbiota in IBS could not be repeatably detected in all researches.

Aside from the microbiota composition in IBS, the causality of microbiome and IBS symptomatology has long been a topic of discussion. An animal experiment by De Palma et al. indicated gut microbiota to be the initiator in some way. The authors administered the fecal microbiota from IBS-D patients and healthy individuals to germ-free mice, respectively. They found that the mice treated with fecal microbiota from IBS-D exhibited anxious behavior and shorter gastrointestinal transit time. This study implied that intestinal microbiota contributed to the symptoms of IBS. Human-based research is required to further illustrate the causal relationship (De Palma et al., 2017). Anyway, a great deal of studies have been assayed in investigating the interactions between microbiota and IBS, whereas most of them only focused on intestinal bacteria, and the term “microbiota” is used nearly congruent with “bacteria.” The role of gut mycobiome also named intestinal fungi in the course of IBS is neglected. Next, we will pose several potential mechanisms of the gut mycobiome influencing the etiology of IBS.

## Putative Roles of Gut Mycobiome in Irritable Bowel Syndrome Pathogenesis

### Mycobiome – Low-Grade Inflammation and Immune Activation – Irritable Bowel Syndrome

The concept of low-grade inflammation and immune activation occurring in IBS patients was first reported in the 1990s and numerous researches confirming this finding sprung up subsequently (Collins, 1992; Spiller and Garsed, 2009; Bashashati et al., 2012). The higher risk of developing IBS after an intestinal infection indicates involvement of aberrant immune reactions in IBS. Enhanced innate immune activity has been observed in the subpopulations of IBS patients, and the role of adaptive immune response in IBS was also confirmed by the altered B-cell activity and elevated amount of T cells (Ohman and Simrén, 2010). Even though there is as yet no direct evidence describing the associations between the gut mycobiome and low-grade mucosal inflammation in IBS, the links are traceable. It is well known that overt pathogenic fungi can lead to various diseases.

The host immune reaction to mycobiome is complex and contains a mass of immune cells and signaling molecules. Host cells of innate immune system and some epithelial cells contain many pattern recognition receptors (PRRs), including C-type lectin receptors (CLRs), Toll-like receptors (TLRs), and so on. PRRs could recognize fungal cell components such as structural polysaccharides of cell wall (β-glucan, chitin, mannan, etc.) and the genetic materials (DNA and RNA; Wang et al., 2014). The innate immune response is activated once PRRs recognizing fungal components, which will drive a series of downstream signaling pathways and then cause antimicrobial and proinflammatory reactions. Subsequently, a myriad of molecules, for instance, immune receptors, cytokines, and chemokines are synthesized, with that the adaptive immune response will be activated to clear fungal pathogens (Netea et al., 2006; Tang et al., 2012). Notably, activated immune system and low-grade inflammation have also been observed in some patients of IBS. In a portion of IBS patients, immune cells like lymphocytes and mast cells are found increased in intestinal biopsies, and inflammatory markers such as IL-1β, TNF-α, IL-6, and IL-8 are elevated (Chadwick et al., 2002; Liebregts et al., 2007; Lazaridis and Germanidis, 2018).

β-glucan is a dominant component of fungal cell wall and can be recognized by Dectin-1, which is a member of CLR family (Brown and Gordon, 2003). Dectin-1 recognizing β-glucan is a meaningful process for facilitating the homeostasis between endogenous mycobiome and host immune responses. When this process is interfered, Dectin-1 can activate downstream signaling pathways to promote cytokine production and inflammatory responses (Iliev et al., 2012). Moreover, a study using a stress-induced hypersensitive rat model demonstrated that β-glucan of fungi can trigger mucosal mast cell degranulation. The more important is that mast cell degranulation is a crucial mechanism of visceral hypersensitivity, which is closely linked to IBS pathogenesis (Larauche et al., 2009; van den Wijngaard et al., 2010). Except for Dectin-1, TLR is another vital member of PRRs detecting fungal microbes in humans (Becker et al., 2015). From the side of IBS, TLRs have been previously reported increased in IBS patients with depression and stress-induced hypersensitive animal models (McKernan et al., 2009; Jizhong et al., 2016). The pathway mediated by TLRs may be another possible mechanism of mycobiome inducing inflammation in IBS. Likewise, dendritic cell is a key immune cell in both of innate and adaptive immunity, and its role in IBS pathogenesis through activating mast cells and inducing visceral hypersensitivity has been emphasized (Long et al., 2012). On the other side, dendritic cells can be modulated by PRRs combining with fungi and induce adaptive immune responses, predominantly helper T-cell response, and then produce cytokines such as IL-17A and IL-22 (Li et al., 2018). Furthermore, the immunogenicity of fungal components can defect the intestinal immunity and disrupt the intestinal mucosal barrier. Collectively, the immunologic reactions to exogenous and endogenous fungi in individuals with IBS lend support to the role of mycobiome in IBS etiopathogenesis. However, more absolute and compelling evidence is required to verify this concept.

### Mycobiome – Fungal-Bacterial Interactions – Irritable Bowel Syndrome

Both mycobiota and bacteria are common members of human intestinal colonizers. Though the abundance of intestinal fungi is less than bacteria by several orders of magnitude, tight interplay between the two microorganisms is essential for maintaining microbial balance in the human gut. Bacterial and fungal dysbiosis could be observed in diverse digestive diseases. The alternation of intestinal microbiota may be a successive effect after fluctuations of either species. Early researches on the interactions of gut fungi and bacteria primarily concentrated on intestinal inflammation and IBD.

The effects of fungal microbes on gut bacteria were explored in a mouse model with dextran sulfate sodium induced colitis. Elevated *Penicillium*, *Wickerhamomyces*, *Alternaria*, and *Candida* together with decreased *Cryptococcus*, *Phialemonium*, and *Wallemia,* and unidentified *Saccharomycetales* were revealed in the inflammatory intestines. Concomitantly, distinct bacterial dysbiosis was found after fungi depleted by fluconazole in these mice. Moreover, the mice with fungal depletion showed aggravated colitis, while the colitis in bacteria-depleted mice exhibited a trend of remission. The authors also proposed that the mycobiota was able to counterbalance to bacterial microbes for keeping the microbial homeostasis in intestines with acute inflammation. Conversely, in chronic recurrent colitis, fungi could exacerbate disease severity and shift into abnormal locations outside the gut (Qiu et al., 2015). Another study based on animal models also revealed the tight relationship between intestinal fungi and bacteria. In this experiment, the authors found that *Enterobacteriaceae* influenced the colonization of fungal species in the gut and indirectly affected the severity of colitis (Sovran et al., 2018).

Sokol et al. also reached a similar conclusion in IBD patients. Detecting fecal microbiota of 235 patients with IBD and 38 healthy individuals revealed a distorted fungal constitution in IBD, as with an elevated *Basidiomycota*/*Ascomycota* ratio. Moreover, positive correlations were observed between several fungal genus with bacterial lineages, such as the association between *Saccharomyces* with some decreased bacteria including *Bifidobacterium*, *Ruminococcus,* and so on (Sokol et al., 2017). A separate study in the same year underscored the interactions between the bacteria and mycobiota in Crohn’s disease. In patients with familial Crohn’s disease, the richness of *Candida tropicalis* elevated significantly and positively related to *Serratia marcescens* and *Escherichia coli*. Besides, the authors also put forward some feasible mechanisms for explaining the interactive relationship. First, sturdy biofilms composed of the components of *Candida tropicalis*, *Escherichia coli,* and *Serratia marcescens* were observed through transmission electron microscopes. Second, there were evident reciprocal roles among interspecies in this biofilm. *Escherichia coli* was closely attached to the cell walls of *Candida tropicalis*; meanwhile, the pili of *Serratia marcescens* acted as a link between *Escherichia coli* and the fungus (Hoarau et al., 2016).

Furthermore, the previous study demonstrated that lipopolysaccharide from bacteria could potentiate the maturation of fungal biofilm (Bandara et al., 2009). In summary, the complex interactions between fungal and bacterial microbes have been verified in IBD and animal models. Notwithstanding no direct evidence pointing out the interaction of gut fungi and bacteria in IBS, altered intestinal mycobiota and bacteria have been discovered in IBS patients. It can be rationally envisaged that gut mycobiome participates in the pathophysiological events of IBS resorting to its interaction with bacterial taxa. For all that, the concrete relations among fungal dysbiosis, fungal-bacterial interactions, and IBS yet need to be intensely studied.

### Mycobiome – Visceral Hypersensitivity – Irritable Bowel Syndrome

Visceral hypersensitivity or enhanced visceral perception is a reduced threshold for pain sense, which has been considered as one of the major pathophysiological features of IBS. Considerable research efforts confirm that visceral hypersensitivity plays a pivotal role in arousing abdominal manifestations like functional abdominal pain in IBS patients. It was reported in former studies that a subset of around 60% of IBS patients is observed by increased visceroperception (Ludidi et al., 2012). Substantial evidence has corroborated the involvement of gastrointestinal microbiota in the pathogenesis of visceral hypersensitivity (Hong and Rhee, 2014; Moloney et al., 2016; Bai et al., 2018). In 2017, a study by Sara Botschuijver and his colleagues firstly reported the associations between the gut mycobiome and visceral hypersensitivity in IBS patients and animal models. In this study, 57 volunteers were tested, including 18 healthy individuals, 19 hypersensitive, and 20 normosensitive IBS patients. Apparent fungal dysbiosis was found in IBS groups rather than in healthy individuals (Botschuijver et al., 2017). Another investigation by the same scientific research team identified gut mycobiome dysbiosis in maternally separated rats again. Moreover, a combination of peppermint and caraway oils, which has antifungal properties, reversed visceral hypersensitivity and changed the composition of gut mycobiome in these animal models (Botschuijver et al., 2018).

*Saccharomyces cerevisiae* and *Candida albicans* were revealed to be the dominant species in both of healthy and IBS groups, whereas the proportion of the two species in IBS patients was much higher than in the healthy. Moreover, the mycobiome signature of hypersensitive IBS patients was distinct from patients with normal sensation. Aside from the investigations of human fecal mycobiome, the authors also further explored the correlations of fungi and visceral hypersensitivity in a hypersensitive rat model. As expected, mycobiome dysbiosis was observed in rats undergoing maternal separation (hypersensitive) in comparison with non-handled rats (normosensitive). Additionally, the study demonstrated that the hypersensitivity of rats, which separated from their mothers, could be reduced to normal levels after being administered with fungicides. More interestingly, transplanting the cecal mycobiome from hypersensitive rats to those normosensitive rats could restore the hypersensitivity of colonic distension. In short, fungal dysbiosis was confirmed existent in IBS patients, and the elimination of fungi could recover the visceral hypersensitivity to normal levels (Botschuijver et al., 2017). In line with this finding, some earlier studies similarly reported yeast-free diets and antifungal treatments to be helpful for IBS subjects (Costabile et al., 2014). As outlined, the associations between disordered mycobiome and visceral hypersensitivity have been investigated, which consolidate the role of mycobiome in IBS etiology. Nevertheless, the present data of human mycobiome can only describe the connections between fungi and IBS, but cannot tell the causal relationships.

### Mycobiome – Gut-Brain Axis – Irritable Bowel Syndrome

It has been acknowledged for a long time that the gut is closely connected with the central nervous system (CNS), as supported by the concept of visceral pain, sense of satiation, and so on. Meanwhile, brain-gut axis also plays a key role in the pathophysiological mechanisms of IBS. For instance, dysregulated hypothalamic-pituitary-adrenal (HPA) axis and altered sympathetic/parasympathetic function were observed in IBS patients by some former researches (Chang et al., 2009; Fukudo, 2013). Also, the most direct evidence for the communication between the brain and gut is the high incidence of neurological symptoms, such as anxiety and depression in IBS sufferers (Thijssen et al., 2010). In recent years, the notion of microbiome-gut-brain axis has emerged rapidly in which the function of gut microbiome was highlighted. To date, studies on microbiome-gut-brain axis mostly focused on the relationships of bacteria and brain-gut axis; however, fungal components and some other minority kingdoms were neglected.

In fact, the gut mycobiome has been reported to participate in the pathogenesis of some psychiatric disorders, and its role in the microbiome-gut-brain axis was concluded in a few of articles. In part of patients with schizophrenia, a fungal dysbiosis was found, as identified by an elevated level in *Candida albicans* and *Saccharomyces cerevisiae* (Severance et al., 2012). Besides, more than double abundance of the *Candida* genus was observed in the autism patient (Strati et al., 2017). What is more, the stool sample from a young woman with severe anorexia nervosa showed an overtly lower diversity of fungi (Gouba et al., 2014). All of the evidence noted supra confirmed the involvement of gut mycobiome in various neurological disorders and prompted the potential role of gut fungi in the microbiome-gut-brain axis. Rett syndrome is a neurological genetic disorder, which reported to be linked to gastrointestinal dysfunctions. Once again, a higher level of *Candida* genus was found in the fecal samples from individuals with Rett syndrome (Strati et al., 2016). In 2016, a case-control cohort analyzing the impacts of *Candida albicans* exposure on cognitive deficits was taken. The increased *Candida albicans* was found to be connected with gastrointestinal dysfunctions and poorer cognitive ability in this study (Severance et al., 2016). Another crucial evidence for the involvement of gut mycobiome in brain-gut axis is that fungal dysbiosis was recognized in IBS patients. Hence, one can see that gut mycobiome is another essential member of the microbiome-brain-gut axis.

Analogous to the role of bacterial microbiota in the brain-gut axis, the communication between the enteric fungi and CNS is a bidirectional system. This mutual communication offered an extra dimension in our understanding of IBS. A review in 2018 proposed that the gut mycobiome appears to share multiple communication pathways with the gut bacterial flora. The assumption assisted in establishing some upstream and downstream pathways for the mycobiome-gut-brain axis in the healthy and disordered status (Enaud et al., 2018). This bidirectional system contains numerous neuro-immuno-endocrine mediators and complex networks among the mycobiome, gut, and CNS. CNS may influence the composition of gut mycobiome through the HPA axis, which is closely associated with cognitive functions and gastrointestinal dynamics and colonic hypersensitivity (Taché et al., 2009; Enaud et al., 2018). In turn, the mycobiomes are capable to synthesize and release substantial mediators to act on the CNS. For example, *Candida albicans* can produce histamine, which is a crucial neuromediator in regulating sleep rhythm, appetite, and cognitive actions (Voropaeva, 2002). As previously reported, the patients with depression exhibited the perturbance in HPA axis with a higher level of cortisol (Penninx et al., 2013). On another side, the gut mycobiome was able to modulate the production of cytokines, such as *Aspergillus fumigatus*, *Candida albicans,* and *Saccharomyces cerevisiae*, which were found to link with increased IL-6 in intestinal mucosa (Czakai et al., 2016; Rizzetto et al., 2016). Additionally, cytokines like IL-1 and IL-6 could activate the HPA axis and then increase the release of cortisol (Dantzer, 2009). Accordingly, it is worth speculating that the aberrant gut mycobiome may be implicated in depression onset, which is a common manifestation in a large proportion of IBS patients. However, the existing findings on the role of the gut mycobiome in brain-gut axis and IBS pathogenesis are not in full accord. Different fungal species in the intestine generate various effects, as some contribute to the disease progression and some specific species may be beneficial for relieving the neurological and gastrointestinal symptoms. Nevertheless, the mycobiome dysbiosis has been confirmed in IBS patients, and the possible role of mycobiome in microbiota-gut-brain axis has also been concluded. Therefore, it could be reasonably hypothesized that the gut mycobiota plays a specific part in the microbiota-gut-brain axis disturbance correlated with IBS.

## Modulation of Gut Mycobiome for Managing Irritable Bowel Syndrome

### Dietary Modification

Diet has consequence for the constitution and metabolic routine of the intestinal microbiome (Xie et al., 2018). For example, high-fat diet promoted the colonization of *Bilophila wadsworthia*, which produced H_2_S and was thought to induce intestinal inflammation (David et al., 2014; Bibbò et al., 2016). Similarly, the mice received food with high fat exerted distinct increases in *Firmicutes* and *Proteobacteria*, and low-grade inflammation was detected in this model (Hildebrandt et al., 2009). Such researches supported the role of diet as one of the determinant factors of microbiota composition. In regard to IBS, most frequently explored diet mode is FODMAPs, which have been observed to provoke symptoms in a subset of IBS patients (Dolan et al., 2018). The supposed mechanism was that some certain food could lead to abnormal gut fermentation with the aspect of altering microbial composition. The recommendation of low FODMAPs in IBS patients is sustained by some clinical data. However, low FODMAPs could also trigger the disturbance in gut microbiota; thus, associations between low FODMAPs and microbiota composition ought to be considered during the application of this diet mode (McIntosh et al., 2017).

Distinct from bacterial composition reacting to the nutrient alternations (e.g., carbohydrates, fiber, and fats), the intestinal fungal constitution seemed to be directly driven by dietary intake (David et al., 2014). It can be indicated that the ingestion of food has great implications on the composition of gut mycobiome. As formerly stated, symptoms of IBS possibly generate from *Candida* overgrowth, and the *Candida* associated diarrhea has similar symptoms with IBS (Wang et al., 2014). What is more, the abundance of *Candida* was reported to be positively associated with near-term carbohydrate intake. Thereby, modulation of the intake of carbohydrate to restrict the *Candida* overgrowth might be an effective strategy for management. This proposed strategy is in accordance with the suggestion of a low FODMAP diet for IBS patients. Furthermore, yeast-eliminated diet could be another beneficial measure for managing IBS. A study on the impacts of different breadmaking processes on *in vitro* parameters in IBS was carried (Costabile et al., 2014). The breads made by the Chorleywood Breadmaking Process, which needed higher percentage of yeasts for fermentation, were more likely to cause IBS associated symptoms than traditional yeast fermentation.

In short, dietary modulation aimed at modifying the intestinal microbiota including bacteria and fungi is speculated to be a useful therapeutic strategy for IBS. On the other hand, individualized advice for modifying diet ought to be considered because different microbial species exhibit distinct responses to dietary changes.

### Probiotics and Prebiotics

Numerous clinical trials on the usage of probiotics or prebiotics in treating IBS have been performed to evaluate their efficacy on the symptoms of IBS sufferers (Cao et al., 2018). An overall beneficial effect of probiotics and prebiotics in relieving IBS was reported in many cases (Ford et al., 2014; Stern and Brenner, 2018). The probiotics tested in IBS mostly belong to *Lactobacillus* or *Bifidobacterium* species, as the two species were deemed as safe in general. Regarding to the mechanisms, some studies based on animal models proposed several possible pathways in favor of the applications of probiotics in IBS. Probiotics can keep the pathogens off through enhancing the intestinal mucosal barrier. Also, probiotics could increase the number of butyrate producing strains (e.g., *Anaerotruncus* and *Faecalibacterium*), and the abundance of butyric and acetic acid in feces increased congruously (Wang et al., 2018). Elevated short-chain fatty acids acidize the intestines and facilitate the colonization of beneficial species including *Lactobacillus*, *Bifidobacterium*, and so on (Johansson et al., 1998). Moreover, some probiotic bacteria were demonstrated to increase the expression of μ-opioid and cannabinoid receptors in intestinal epithelium and thereby reduce colonic hypersensitivity (Rousseaux et al., 2007). Modulating immune functions and reducing inflammation are also supporting evidence for the beneficial roles of probiotics in IBS (O’Mahony et al., 2005). Prebiotics are nondigestible food ingredients enhancing the proliferation or colonization of healthy intestinal microbiota (Gallo et al., 2016). The mechanisms are supposed to be anti-inflammation, inhibiting the adherence of pathogen and so on. Prebiotics also have influence on the composition of gut microbiota. For instance, IBS patients who were treated with a trans-galactooligosaccharide mixture showed a significant relieve in symptoms, and the proportions of *Bifidobacterium* were apparently increased (Silk et al., 2009).

In addition to the roles noted above, influencing the intestinal fungal constitution may be another vital function of probiotics or prebiotics. Take the probiotics as an example, two strains of probiotic lactobacilli were reported to have apparent antagonistic effects against *Candida glabrata*, which is closely associated with vulvovaginal candidiasis (Chew et al., 2015a,b). This indicated the antifungal properties of some probiotic strains. What is more, a fungal probiotic strain-*Saccharomyces boulardii* was demonstrated to be effective for improving symptoms and the quality of life of IBS patients (Abbas et al., 2014). As well in IBS animal models, *Saccharomyces boulardii* was found useful in alleviating gastrointestinal dysfunctions (Brun et al., 2017). On another hand, administration of *Saccharomyces boulardii* in preterm infants was reported helpful to disrupt fungi colonization and prevent invasive fungal infections (Demirel et al., 2013). Despite no study has directly analyzed the influence of probiotics on gut mycobiome till now, the probiotics having impacts on fungal microbes have been reported. This is likely to be an additional supporting theory for treating IBS with probiotics and prebiotics, whereas enormous studies yet need to be taken for more valid proofs.

Despite there are numerous evidence in support of the beneficial roles of probiotics and prebiotics, different voices can be heard on this issue. Recently, two emerging papers of *Cell* challenged the efficiency of probiotics to some extent. Zmora et al. found that probiotics are resisted by indigenous microbiome in mice intestines, and similarly in humans, person-specific resistance to the colonization of probiotics is observed as well (Zmora et al., 2018). Moreover, another research by Jotham Suez and colleagues proposed that probiotics cause a delay in gut microbiome restoring to the pre-antibiotic state (Suez et al., 2018). Actually, this is not the first time that negative studies for probiotics were put forward. For example, a clinical trial on *Bifidobacterium longum NCC3001* relieving depression in IBS patients was also condemned to be fragile (Meyer and Vassar, 2018). As to prebiotics, failing to relieve symptoms of IBS patients was reported too (Wilson et al., 2019). Results like these prompt that the effectiveness and clinical application of probiotics and prebiotics still need further verification. Nonetheless, the functions of probiotic strains should not be negated thoroughly according to the existing data. Researches on individualized regimens, large-scale clinical trials, and efficacy of single strain are all in demand.

### Fecal Microbiota Transplantation

Fecal microbiota transplantation (FMT) is the process of transferring the microbiota from healthy donors to the patients with gastrointestinal dysbiosis, thereby replacing or strengthening the composition of intestinal microbiota in recipients (König et al., 2017). Up to now, in clinical practice, administering FMT to the patients with *Clostridium difficile* induced diarrhea is the most intensely recommended (van Nood et al., 2013). Take a recent randomized controlled trial in Norway as an example, the proportion of symptom relief was found to be significantly higher in the FMT groups than in the controls (Johnsen et al., 2018). Except from symptoms relief, some other cases reported the improvements in quality of life in the patients with IBS after FMT (Holvoet et al., 2017). On balance, FMT is supposed to be effective for managing IBS to a certain degree, but stronger evidence is in requirement. To our knowledge, the fecal microbiota from a healthy donor is the natural mixture of the human intestinal microbiota. The administration of xenogenous microbiota is bound to alter the microbial constitution in the recipients, including the composition of bacteria, fungi, protozoa, and so on. A recent study on the efficacy of FMT in *Clostridium difficile* infection analyzed the intestinal fungi in recipients. In this study, the researchers observed that successful FMT recipients displayed more *Saccharomyces* and *Aspergillus* in intestine; conversely, non-responders showed higher proportion of *Candida*. More *Candida albicans* in donor feces were found to link with reduced effectiveness of FMT (Zuo et al., 2018). The analysis of intestinal fungi in IBS patients treated with FMT is another meaningful topic for future studies. Actually, IBS patients treated with FMT did not always come up with a good ending. For example, a placebo-controlled research launched by Halkjær et al. reported that IBS patients in the FMT group did not experience better symptom relief than the placebo group (Halkjær et al., 2018). In addition, more well-designed and larger studies are requested for drawing a consistent conclusion on the role of FMT in treating IBS.

### Stool Frequency

Stool frequency is one of the common indicators to evaluate IBS symptoms. Bowel movement and the intestinal microbiome are explicitly related. A population-based cohort study reported the direct connections between stool frequency and gut microbiota (Hadizadeh et al., 2017). Mainly, *Bacteroides*/*Firmicutes*, *Bacteroides*/*unclassified_Ruminococcaceae,* and *Bacteroides* are positively correlated with stool frequency, while *unclassified_Ruminococcaceae*, *Alistipes*, *Oscillobacter*, and so on presented negative correlation. The causal relationship of gut motility and microbiome has yet been clearly investigated, and the interactional relation is more recommended. From one side, stool frequency is a vital modulator of gut microbiome; in other words, intestinal motility shapes the composition of resident microbiota. For instance, mice treated with polyethylene glycol exert apparent short intestinal transit time, and the levels of order *Bacteroidales* and family *Bacteroidaceae* showed a significant increase. Conversely, intestines with slower motility inhabit more *Porphyromonadaceae*. Moreover, some clinical disorders, which are commonly accompanied with impaired motility (such as small bowel bacterial overgrowth), also support the point that altered bowel movement influencing the microbiota composition. From the other side, the microorganisms in digestive tracts are able to modulate the secretion of neurotransmitters and hormones, including serotonin, gastrin, and so on, which subsequently affect the gastrointestinal motor function (Kashyap et al., 2013).

Up till now, the researches of stool frequency and gut microbiome centered on bacterial species, whereas fungal microbes were neglected. A pilot study found that a fermentate from *Saccharomyces cerevisiae* could improve gastrointestinal transmission function *via* modulating microbiome (Pinheiro et al., 2017). As *Saccharomyces cerevisiae* is one of the resident populations in human gut, this result reflected the interactions among bacteria, fungi, and gut motility to some degree. Even so, direct evidence proving the mutual connections between gut mycobiome and stool frequency is scanty to date. The modulation of gut motility on mycobiome is another meaningful topic that demands deeper research.

## Perspectives

Intestinal microbiota plays an essential role in both the pathogenesis and management of IBS. The intestinal fungi, namely gut mycobiome, have been neglected for a long term. In the present review, we summarized the potential roles of gut mycobiome in the etiology and therapy of IBS, which help broaden the knowledge of the intestinal microorganism and catapult fungal species to the center stage in the future exploration of gastrointestinal diseases. By far, the study on the contribution of gut mycobiome to IBS is yet in its infancy, and numerous problems remain to be addressed. For instance, the associations between gut mycobiome with the classification, differential diagnosis, and relapse of IBS are all subjects that merit further study. The summary of the current researches on mycobiome and IBS would provide an additional dimension to the investigation of microbiota in digestive diseases. Furthermore, better understanding of the gut mycobiome in IBS will provide new treatment options and ameliorate the quality of life for IBS patients.

## Author Contributions

YG, GZ, and HC contributed to conception and design of the study. XQ and SH searched literature and wrote sections of the manuscript. XQ, BW, and HC organized the framework and revised the manuscript. All authors contributed to manuscript revision, read, and approved the submitted version.

### Conflict of Interest Statement

The authors declare that the research was conducted in the absence of any commercial or financial relationships that could be construed as a potential conflict of interest.
